# Necrotic Lesions Following Elective Urological Surgery in an Infant

**DOI:** 10.1055/s-0038-1668112

**Published:** 2018-08-08

**Authors:** María Fernández-Ibieta

**Affiliations:** 1 Pediatric Surgery Department, Hospital Clínico Universitario Virgen de la Arrixaca, Murcia, Spain

**Keywords:** necrotizing soft tissue infection, necrotizing fasciitis, surgical fomite, pediatrics, pediatric urology, vacuum-assisted therapy

## Abstract

**Case Report** An 11-month-old female infant presented on the first postoperative (PO) day following an elective pyeloplasty, a dark bluish erythema of her lumbotomy wound, plus a satellite lesion of the same characteristics. Fever and sepsis developed, and despite broad spectrum antibiotics (meropenem and vancomycin) were started, a diagnosis of necrotizing soft-tissue infection (NSTI or necrotizing fasciitis) was established. Surgical debridement of both lesions was performed on day 3 PO, and a surgical contamination (ring retractor blade) was suspected, due to the particular geography of the lesion. Urine and blood cultures yielded no bacteria, but tissue culture grew *Pseudomonas aeruginosa* . At PO 6th day, lesions still appeared exudative and poorly perfused, so vacuum-assisted therapy (VAT) treatment was started. Exudate control, perfusion, and granulation improved in consecutive days, which permitted direct closure (no graft needed) at PO day 12.

**Discussion** *P. aeruginosa* can be a fatal cause of type I NSTI. It has been reported rarely in adult series, with a prevalence of 4%, but it can be a major pathogen in pediatric NSTI. Added to an early recognition, aggressive surgery and debridement are required, in combination with antibiotic therapy, to limit the spread of the infection. In our case, despite surgical debridement being performed on day 3 PO, both wounds maintained scarce perfusion, and debris and exudate were poorly controlled with usual silver foams and daily nursery cures. VAT pediatric device was then added, which rapidly improved surgical bed, enhancing tissue perfusion and granulation in the following days.

## Case Report

**Fig. 1:**
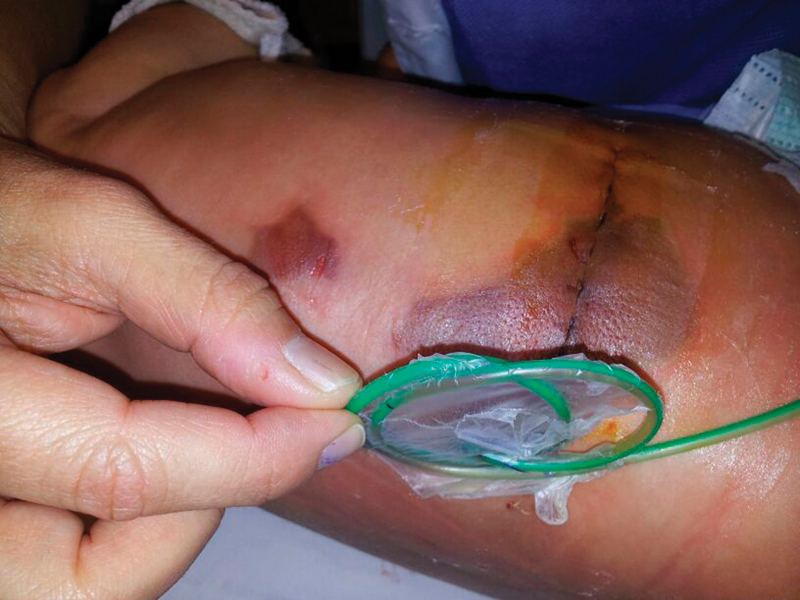
Necrotic lesions in the first operative day. Necrotic bluish lesion on surgical right lumbotomy, and a satellite lesion with similar characteristics. Nephrostomy green catheter on place.

**Fig. 2:**
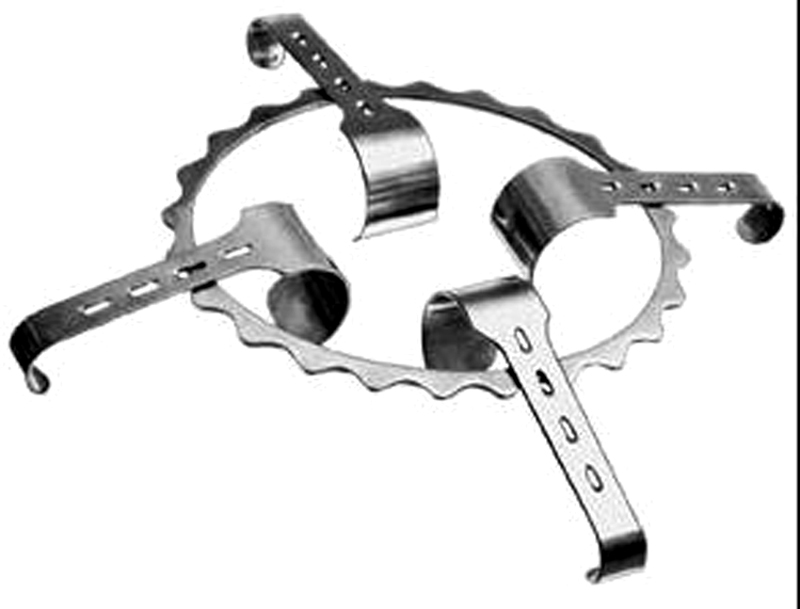
Denis Browne retractor rings and valves. Metallic ring retractor, frequently used in open lumbotomy. Blades commonly lay on unprotected skin.

**Fig. 3:**
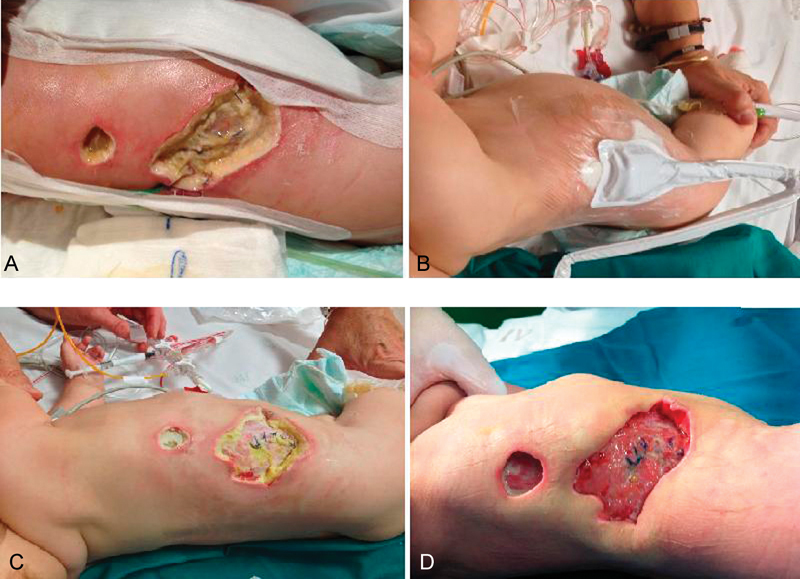
Wound evolution after debridement. ( **A** ) 48 hours after debridement, still showing edge necrosis and exudation. ( **B** ) Vacuum-assisted therapy (VAT) pediatric device on place, at postoperative (PO) day 6. ( **C** ) PO day 8, on VAT treatment, with much less exudation and debris. ( **D** ) Significant improvement on PO day 12, and enhanced granulation.

Following an elective open pyeloplasty for pelvic ureteric junction obstruction, an 11-month-old female presented with low grade fever and irritability on the first postoperative (PO) day. The birth and developmental history were unremarkable, except for two episodes of pyelonephritis and a right pyeloureteral stenosis, which led to a programmed open pyeloplasty. She had been on fosfomycin antibiotic prophylaxis for the last months, and a single dose of amoxiclavulanate was given intravenously during surgery. Examination on the first 12 hours following surgery revealed a febrile irritable infant, with pulse of 120 and normal blood pressure. Neutrophil count (19.8 × 10 ^9^ /L) and C-reactive protein were elevated (9.1 U/L), and her hemoglobin was decreased following surgery (from 11.5 to 8.3 mg/dL). Her surgical wound was by then only slightly erythematous. She was started on cefotaxime therapy (150 mg/kg/day e.v.), and a erythrocyte transfusion was ordered. Nevertheless, the local examination at the next day revealed a dark bluish erythema of the entire surgical wound measuring 9 × 6 cm, plus a satellite lesion of the same characteristics 5 cm away ( Fig. 1 ). These lesions were tender and warm in palpation. As a ring retractor was used during surgery, to enhance surgical field view, and its blades lay directly on peri-incisional skin ( Fig. 2 ), a surgical fomite or contact vehicle mechanism was suspected to explain the satellite lesion. Apart from these, all her right flank appeared also erythematous and warm. C-reactive protein and leukocyte count were raised to 23.5 × 10 ^9^ / L and 23.4 U/L, respectively. Although still febrile, and tachycardic, she needed neither further homodynamic nor ventilatory support. A diagnosis of necrotizing soft-tissue infection (NSTI) with sepsis was determined, and meropenem (60 mg/kg/day) plus vancomycin (40 mg/kg/day) were started. Urine and blood cultures yielded no bacteria. Although fever disappeared the next day (PO 2nd day), the wound worsened, showing blisters on a well demarcated necrotic apparent patch of 9 × 5 cm. The satellite lesion (of 2 × 2 cm) developed in the same manner. On PO day 3, a surgical incision and debridement of both lesions were performed. Necrosis reached the external oblique muscle fascia, and a small fasciotomy was required. Tissue culture grew *Pseudomonas aeruginosa* sensitive to meropenem, and no anaerobic bacteria grew on the corresponding culture. Apart from the above-mentioned antibiotic regimen, daily wound care with silver-containing foam was continued following debridement. At PO 6th day, both lesions still appeared considerably exudative and poorly perfused ( Fig. 3A ), so vacuum-assisted therapy (VAT) treatment was started ( Fig. 3B ). From then on, exudate control, perfusion, and granulation improved in the consecutive days ( Fig. 3C and D ), which permitted direct closure (no grafting needed) at PO day 12. Antibiotics were discontinued and the patient discharged at PO day 15.

## Discussion

Necrotizing fasciitis is a rare, rapidly progressive, and potentially fatal infection of the subcutaneous cellular tissue and muscular fascia. It is characterized by a massive destruction of the tissue and usually accompanied by systemic signs of toxicity. The predisposing factors are hematological malignancy, diabetes mellitus, and infancy in the majority of the cases. 1 2 3 The definition and nomenclature is still debated, so a wider term is now advocated: *NSTI* . 3 4 Rapid aggressive treatment invariably includes surgical debridement. There are four main groups of NSTI, depending on the microbiology. 3 5 Type I NSTI typically affects patients with several comorbidities and is polymicrobial (Gram-positive cocci, enterococci, and Gram-negative Enterobacteriaceae). Type II includes monomicrobial infections caused by β-hemolytic *Streptococcus pyogenes* , sometimes associated with *Streptococcus aureus* . Type III includes monomicrobial infections involving the *Clostridium* species or Gram-negative bacteria. Finally, type IV is the result of fungal infections, mainly *Candida* spp. and zygomycetes. This type is found mainly in the immunocompromised host.

*P. aeruginosa* can be a fatal cause of type I NSTI. It has been reported rarely in adult series, with a prevalence of 4%, 2 but contrary to that, and according to recent reports, it can be a major pathogen in pediatric NSTI. 6 7 8 Moreover, localization of the necrotizing infection in the trunk is rare in adults, but again, not so rare in pediatric series. 6 7 8 There are no large series on pediatric NSTI, due to its rarity, but small series can be found in the literature. Contrary to what is expected, children who develop NSTI are mostly not immunocompromised, and predisposing factors may vary from varicella infections, onfalitis, and dental abscess to streptococcal toxic shock syndrome. 6 But, if immunocompromised, sequelae and mortality rise. Most infections in children are polimicrobial, but *P. aeruginosa* is the most frequently isolated bacteria. Mortality in children reaches 18% according to some series. 6 7 8

In our patient, the peculiar geography of both lesions, the main surgical necrotizing wound and the satellite necrosis, made us suspect of a surgical infection while in the operation theater, due to a probably contaminated surgical fomite: the metallic ring retractor used during the surgical procedure. This was not proven because the valves and retractors were already sterilized when the infection was defined. Nevertheless, we have to point out that this possibility (surgical theater fomites contamination), although very rare, does not have to be disregarded.

Added to an early recognition, aggressive surgery and debridement are usually required, in combination with antibiotic therapy, to limit the spread of the infection. In our case, despite surgical debridement was performed on day 3 PO, both wounds maintained scarce perfusion, and debris and exudate were poorly controlled with usual silver foams and daily nursery cures. VAT pediatric device was then added, which rapidly improved surgical bed, enhancing tissue perfusion and granulation in the next 5 days. Hyperbaric therapy, another possible coadjuvant therapy, 1 2 4 was not used in our case due to the lack of experience in pediatric cases. Contrary to that, VAT pediatric devices are widespread and numerous series 9 10 back its beneficial effects and the feasibility of the VAT even in the youngest patients.
